# Cachexia, dietetic consultation, and survival in patients with pancreatic and periampullary cancer: A multicenter cohort study

**DOI:** 10.1002/cam4.3556

**Published:** 2020-10-27

**Authors:** Anouk E. J. Latenstein, Willemieke P. M. Dijksterhuis, Tara M. Mackay, Sandra Beijer, Casper H. J. van Eijck, Ignace H. J. T. de Hingh, I. Quintus Molenaar, Martijn G. H. van Oijen, Hjalmar C. van Santvoort, Marian A. E. de van der Schueren, Judith de Vos‐Geelen, Jeanne H. M. de Vries, Johanna W. Wilmink, Marc G. Besselink, Hanneke W. M. van Laarhoven, Dutch Pancreatic Cancer Group

**Affiliations:** ^1^ Department of Surgery Cancer Center Amsterdam Amsterdam UMC University of Amsterdam Amsterdam the Netherlands; ^2^ Department of Medical Oncology Cancer Center Amsterdam Amsterdam UMC University of Amsterdam Amsterdam the Netherlands; ^3^ Department of Research & Development Netherlands Comprehensive Cancer Organisation (IKNL) Utrecht the Netherlands; ^4^ Department of Surgery Erasmus MC Rotterdam the Netherlands; ^5^ Department of Surgery Catharina Hospital Eindhoven the Netherlands; ^6^ Department of Surgery Regional Academic Cancer Center Utrecht St Antonius Hospital Nieuwegein and University Medical Center Utrecht Cancer Center Utrecht the Netherlands; ^7^ Department of Nutrition and Health HAN University of Applied Sciences Nijmegen the Netherlands; ^8^ Department of Internal Medicine Division of Medical Oncology GROW ‐ School for Oncology and Developmental Biology Maastricht University Medical Center Maastricht the Netherlands; ^9^ Division of Human Nutrition and Health Wageningen University Wageningen the Netherlands

**Keywords:** cachexia, dietetic consultation, nutritional interventions, pancreatic cancer, weight loss

## Abstract

It is unclear to what extent patients with pancreatic cancer have cachexia and had a dietetic consult for nutritional support. The aim was to assess the prevalence of cachexia, dietitian consultation, and overall survival in these patients. This prospective multicenter cohort study included patients with pancreatic cancer, who participated in the Dutch Pancreatic Cancer Project and completed patient reported outcome measures (2015–2018). Additional data were obtained from the Netherlands Cancer Registry. Cachexia was defined as self‐reported >5% body weight loss, or >2% in patients with a BMI <20 kg/m^2^over the past half year. The Kaplan–Meier method was used to analyze overall survival. In total, 202 patients were included from 18 centers. Cachexia was present in 144 patients (71%) and 81 of those patients (56%) had dietetic consultation. Cachexia was present in 63% of 94 patients who underwent surgery, 77% of 70 patients who received palliative chemotherapy and 82% of 38 patients who had best supportive care. Dietitian consultation was reported in 53%, 52%, and 71%, respectively. Median overall survival did not differ between patients with and without cachexia, but decreased in those with severe weight loss (12 months (IQR 7–20) vs. 16 months (IQR 8–31), *p* = 0.02), as compared to those with <10% weight loss during the past half year. Two‐thirds of patients with pancreatic cancer present with cachexia of which nearly half had no dietetic consultation. Survival was comparable in patients with and without cachexia, but decreased in patients with more severe weight loss.

## Graphical abstract

The extent of cachexia and dietetic consultation in patients with pancreatic cancer in the real‐world setting is unclear. This prospective multicenter cohort study found that in patients, diagnosed between 2015 and 2018, two‐third presented with cachexia of whom nearly half had no dietetic consultation. Survival was comparable in patients with and without cachexia, but decreased in patients with more severe weight loss.

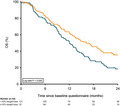

## 1 INTRODUCTION

Patients with pancreatic ductal adenocarcinoma and periampullary carcinoma (hereafter, pancreatic cancer) frequently present with malnutrition.^1^Weight loss or even cachexia is reported in up to 80% of patients with pancreatic cancer.^2^, ^3^, ^4^Weight loss could be caused by reduced dietary intake due to anorexia, abdominal pain, nausea, diarrhea, catabolic effects of the tumor, exocrine, and endocrine pancreatic insufficiency, and duodenal obstruction.^5^, ^6^Cachexia is defined as weight loss greater than 5%, or weight loss greater than 2% in individuals with a low body mass index (BMI, <20 kg/m^2^) or low skeletal muscle mass (sarcopenia) during the past 6 months.^7^Cachexia is nowadays regarded to as the ultimate form of disease‐related malnutrition, whereby, severe malnutrition is defined as a weight loss of 10% or more.^8^, ^9^In pancreatic cancer, severe weight loss is associated with reduced survival, progressive disease, reduced treatment tolerance, and a decrease in quality of life.^2^, ^7^In patients with resectable cancer, it was shown that preoperative weight loss was associated with a shorter survival.^10^This emphasizes the need for early screening (the risk of) for malnutrition and to start nutritional interventions, preferably before start of anticancer treatment, such as surgery.^8^, ^11^, ^12^, ^13^, ^14^, ^15^, ^16^Dietitians can create an individualized treatment plan based on patients’ specific needs, for example, in the context of exocrine and/or endocrine insufficiency or nutritional impact symptoms. Currently, it is unclear to what extent patients with pancreatic cancer in a real‐world setting have cachexia and had a dietetic consult for nutritional assessment and support. Therefore, the aims were to assess the prevalence of cachexia, dietetic consultation, and overall survival in these patients.

## 2 MATERIALS AND METHODS

### 2.1 Study design

This prospective multicenter cohort study included patients with pancreatic cancer who participated in the Dutch Pancreatic Cancer Project (PACAP).^17^PACAP was established in 2013 and includes a collection of clinical and patient reported outcome measures (PROMs, questionnaires) data. Currently, 48 centers participate in this nationwide project (www.pacap.nl). The PROMs data included self‐reported nutritional parameters and body weight. Patient, tumor, and treatment data were retrieved from the Netherlands Cancer Registry (NCR). The NCR is a population‐based registry in which all patients with a newly diagnosed malignancy are identified by automatic notifications of the national pathological archive (PALGA) and the National Hospital Discharge Register. Data within the NCR were collected from medical records by trained NCR administrators. The data from the PROMs and NCR were linked as all patients provided written informed consent for participation and linkage of data. This study was performed in accordance with the STROBE guidelines.^18^

### 2.2 Study population

All patients with pancreatic and periampullary cancer who participated in the PACAP PROMs registry between January 2015 and February 2018 were included. Patients were excluded if the questionnaire was not completed at baseline or if data on weight loss were lacking in any questionnaire.

### 2.3 Data collection

The NCR includes clinical data, specifically sex, age, World Health Organization (WHO) performance status, comorbidities, tumor location, tumor stage, and treatment details regarding resection and chemotherapy. Tumor stage was classified according to TNM 7 for patients diagnosed in 2015–2016 and TNM 8 if diagnosed in 2017–2018.^19^, ^20^Tumor stage was based on the pathological TNM stage. If patients received neoadjuvant therapy or if pathological TNM stage was not available, clinical TNM stage was used. Patients were categorized into three groups based on treatment (a) patients who underwent surgery (pancreatoduodenectomy, other pancreatectomy, or irreversible electroporation or radiofrequency ablation) and regardless of receiving or completing (neo)adjuvant chemotherapy; (b) patients who started palliative chemotherapy (patients who started neoadjuvant chemotherapy but did not undergo surgery were also included) and; (c) patients who received best supportive care. PROMs were collected at baseline and at 3, 6, 9, 12, 18, and 24 months after baseline, and yearly thereafter, until death or dropout. Results from baseline, 3, and 9 months were used, because dietary intake measurements (by the Dutch Healthy Diet Food Frequency Questionnaire (DHD‐FFQ)) were available at these time points. A questionnaire was considered a baseline measurement if the first questionnaire was completed (best supportive care group) or if it was completed between the date of diagnosis and (a) date of surgery or (b) date of start of neoadjuvant or palliative chemotherapy or within 1 week after start of chemotherapy (i.e. considering that this would not influence the results, because most questions were about a longer retrospective period). Survival data were obtained by linkage with the Municipal Personal Records Database which contains the vital status of all Dutch inhabitants. Overall survival time was defined as the time between date of diagnosis and date of death or censoring (1 February 2020).

### 2.4 Nutritional status

The PROMs contained questions on the following domains with respect to nutritional status and interventions: height, current weight, weight loss, dietetic consultation (including both intramural and extramural health care), self‐reported reduced food intake, appetite, use of oral nutritional supplements or parenteral nutrition, and tube feeding. BMI (kg/m^2^) was calculated from reported weight and height. A dietetic consultation is designed in accordance the Dutch Guidelines on malnutrition.^21^A consult starts with screening of and diagnosing malnutrition and, in case of malnutrition, an extensive nutritional assessment will be performed. Based on these results, an appropriate and individualized treatment plan will be made. Cachexia was defined as >5% body weight loss, or >2% with a BMI of <20 kg/m^2^according to the international consensus criteria of Fearon et al. (2011).^7^Severe weight loss (≥10%) was calculated from the reported body weight and weight loss over the past half year before diagnosis in the PROMs. The DHD‐FFQ was completed by patients who consumed food orally. This is a validated questionnaire based on national dietary guidelines including questions about portion sizes of bread, dairy, meat, fish, vegetables and candy, and alcohol consumption.^22^, ^23^Based on previously described methods, a protein score was calculated from the results of the DHD‐FFQ ranging from 0 to 10 and categorized into two categories: 0–9.9 or 10.^22^, ^24^A score <10 corresponds with a protein intake that provides room for improvement by increasing the consumption of protein sources that were included in the DHD‐FFQ for the general healthy population.^23^Patients with cancer have higher protein requirements than healthy persons, and therefore, a protein intake assessed as needed to be improved for a healthy person could be interpreted as needed for dietetic consultation and/or oral nutritional supplements for cancer patients.

### 2.5 Statistical analysis

Baseline characteristics and nutritional parameters were presented using descriptive statistics. Normally distributed continuous data were presented as means with standard deviations (SD). Non‐normally distributed continuous data were presented as medians with interquartile ranges (IQR). Categorical data were presented as frequencies with percentages. Median overall survival was calculated using the Kaplan–Meier method in patients with and without cachexia, and with <10% and ≥10% weight loss at baseline. Overall survival was compared using the log‐rank test. *p*‐values <0.05 were considered statistically significant. Analyses were performed using SAS software (version 9.4, SAS institute).

## 3 RESULTS

**FIGURE 1:**
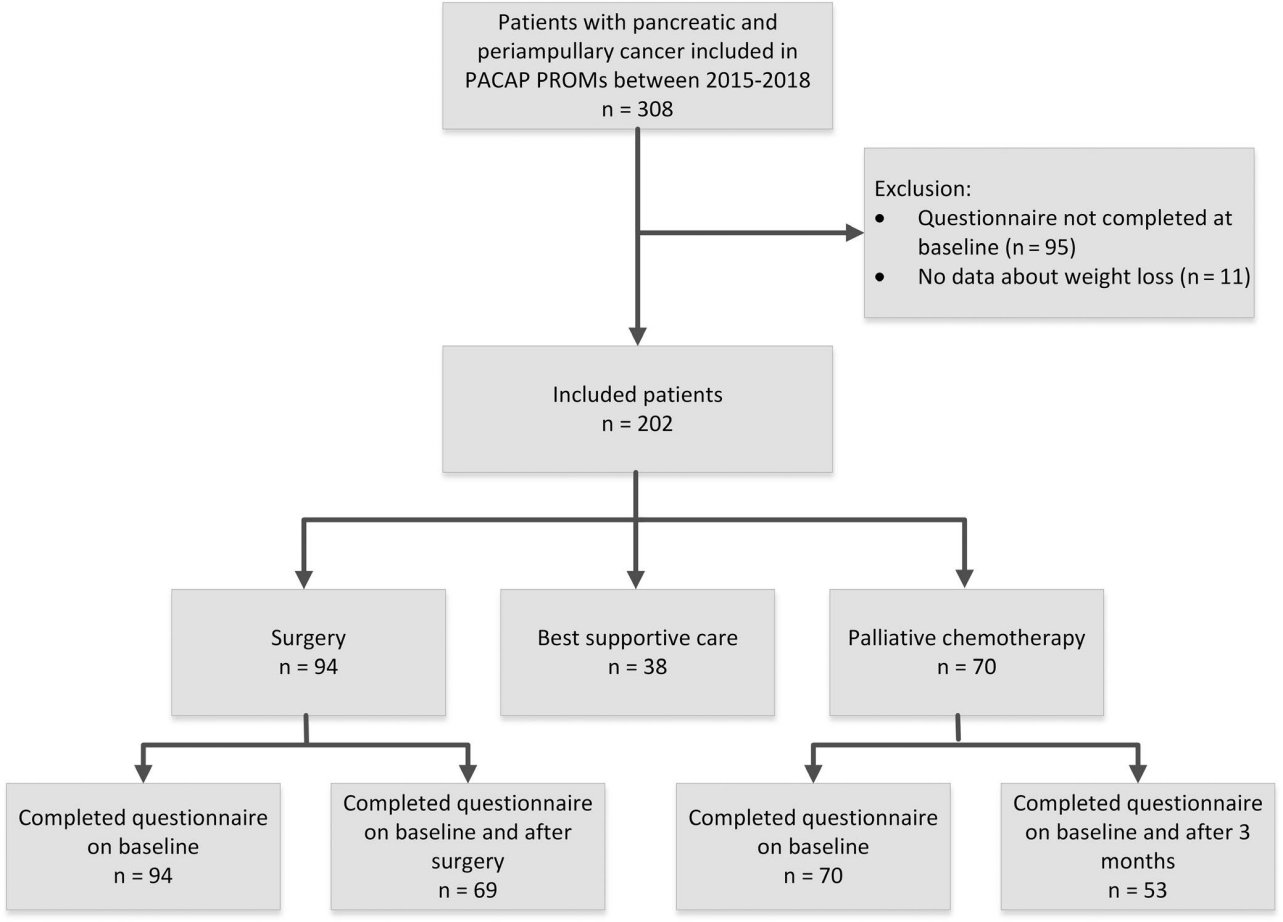
Flow diagram of patient inclusion

In total, 308 patients with pancreatic cancer from 18 centers were included in the PACAP cohort between 2015 and 2018 (Figure 1). Of these patients, 95 were excluded because the first questionnaire was not completed at baseline and 11 because data about weight loss were lacking. Of the remaining 202 patients, 94 (47%) were female and the median age was 68 years (IQR 62–73, Table 1). The majority of patients (n = 132, 65%) had a WHO performance status of 0 or 1. Of the 94 patients (47%) with surgery, 79 (84%) underwent a pancreatoduodenectomy and 57 (61%) received neoadjuvant and/or adjuvant chemotherapy. Neoadjuvant therapy had a median duration of 50 days (IQR 43–91). Seventy out of the 202 patients (35%) received palliative chemotherapy, and 38 patients (19%) received best supportive care.

**TABLE 1:** Baseline characteristics of all patients and stratified per type of treatment

	All patients (n = 202)	Surgery (n = 94)	Palliative chemotherapy (n = 70)	Best supportive care (n = 38)
Female, No. (%)	94 (47%)	42 (45%)	35 (50%)	17 (45%)
Age, years, median (IQR)	68 (62–73)	70 (62–74)	65 (60–70)	72 (62–77)
<55	17 (8%)	8 (9%)	6 (9%)	3 (8%)
55–64	60 (30%)	25 (27%)	25 (36%)	10 (26%)
65–74	83 (41%)	40 (43%)	33 (47%)	10 (26%)
≥75	42 (21%)	21 (22%)	6 (9%)	15 (39%)
Performance status, No. (%)				
0 or 1	132 (65%)	60 (64%)	52 (74%)	20 (53%)
≥ 2	21 (10%)	4 (4%)	7 (10%)	10 (26%)
Missing	49 (24%)	30 (32%)	11 (16%)	8 (21%)
Comorbidities, No. (%)				
0	45 (22%)	19 (20%)	17 (24%)	9 (24%)
1	66 (33%)	35 (37%)	22 (31%)	9 (24%)
≥2	70 (35%)	30 (32%)	24 (34%)	16 (42%)
Missing	21 (10%)	10 (11%)	7 (10%)	4 (11%)
Tumor location, No. (%)				
Pancreas	163 (81%)	63 (67%)	64 (91%)	36 (95%)
Periampullary	39 (19%)	31 (33%)	6 (9%)	2 (5%)
Clinical stage, No. (%)				
I	27 (13%)	14 (15%)	6 (9%)	7 (18%)
II	46 (23%)	32 (34%)	11 (16%)	3 (8%)
III	72 (36%)	43 (46%)	19 (27%)	10 (26%)
IV	55 (27%)	3 (3%)	34 (49%)	18 (47%)
Missing	2 (1%)	2 (2%)	0 (0%)	0 (0%)
Surgery, No. (%)				
Pancreatoduodenectomy	79 (39%)	79 (84%)	NA	NA
Other pancreatectomy	11 (5%)	11 (12%)	NA	NA
IRE/RFA	4 (2%)	4 (4%)	NA	NA
Chemotherapy, No. (%)				
Neoadjuvant only	5 (2%)	5 (5%)	NA	NA
Adjuvant only	42 (21%)	42 (45%)	NA	NA
Neoadjuvant and adjuvant	10 (5%)	10 (11%)	NA	NA
Palliative	70 (35%)	NA	70 (100%)	NA

Abbreviations: BMI, body mass index, IQR, interquartile range; IRE, irreversible electroporation; NA, not applicable; RFA, radiofrequency ablation.

### 3.1 Nutritional status

At diagnosis, cachexia was present in 144 patients (71%). In 81 of these cachectic patients (56%), dietetic consultation was reported at baseline, compared to 7 patients in the group without cachexia (12%). At baseline, 40% of all included patients (n = 81) presented with ≥10% weight loss during the past 6 months and 52 of these patients (64%) had dietetic consultation. The protein score (<10) was insufficient in 147 patients (78%), of whom 58 patients (39%) were seen by a dietician.

**FIGURE 2:**
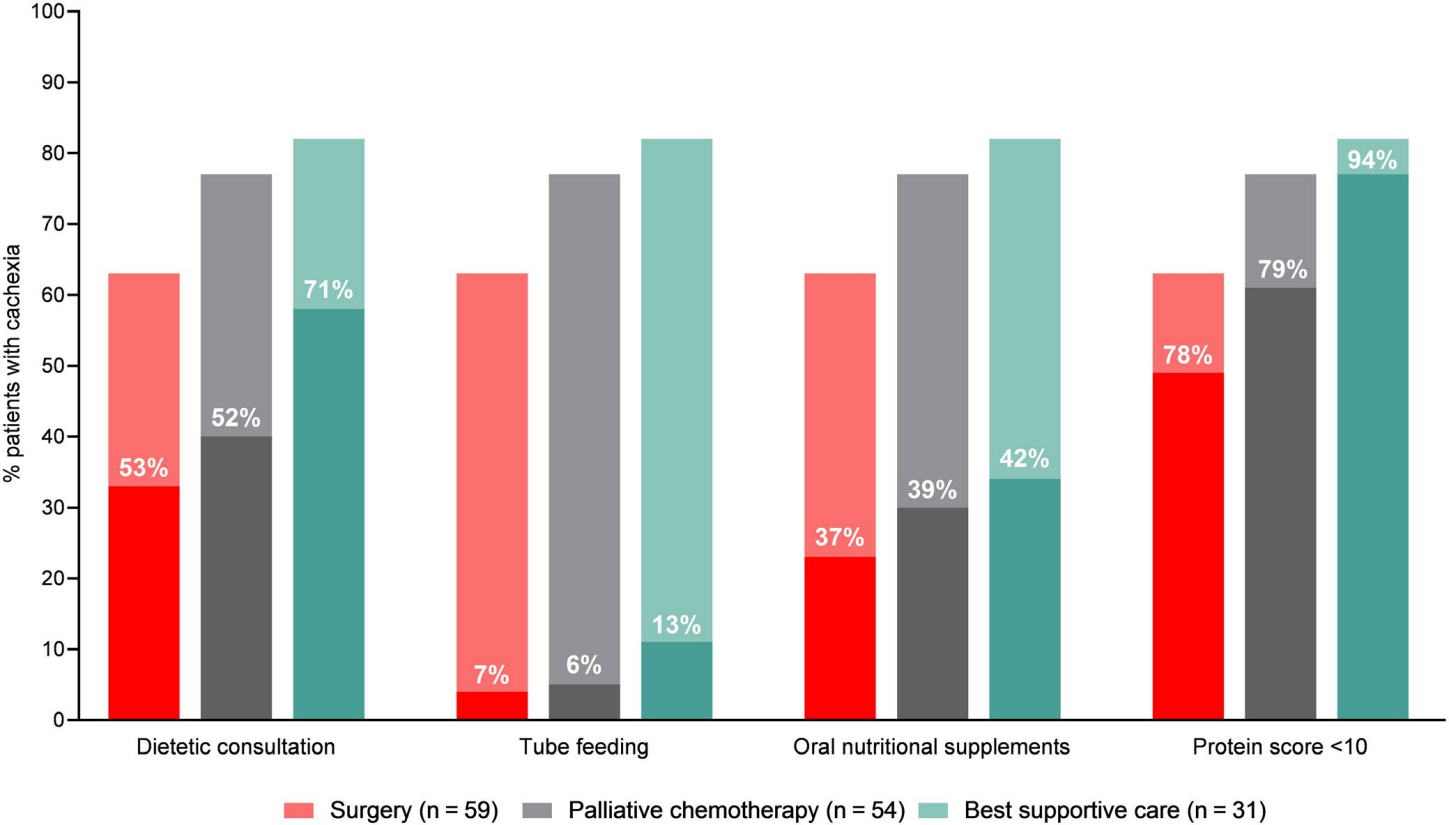
Nutritional parameters in patients with cachexia at baseline.Percentages within the bars reflect the proportion of cachectic patients that had a dietitian consultation, received a nutritional intervention, or had a protein score <10

Of all 94 patients who underwent surgery, 59 patients (63%) had cachexia at baseline of whom 31 (53%) had a dietetic consultation (Table 2, Figure 2). The presence of cachexia did not differ between patients with a BMI <25 compared to those with a BMI ≥25 (35 patients (69%) vs. 29 patients (69%), respectively, *p* = 0.965), as accounted for weight loss ≥10% (17 patients (33%) vs. 11 patients (26%), respectively, *p* = 0.455). At baseline, 4 patients (4%) received tube feeding, 28 patients (30%) used oral nutritional supplements, and none used parenteral nutrition. The protein score was insufficient at baseline in 62 patients (66%), 23 of these patients (37%) had dietetic consultation, and 21 patients (34%) received oral nutritional supplements. Pancreatic enzyme supplementation was given to 13 patients (19%) at baseline and to 44 patients (64%) after surgery.

**TABLE 2:** Nutritional and weight parameters in patients who underwent surgery

	Patients who completed the questionnaire at baseline^a^(n = 94)	Patients who completed a questionnaire at baseline and after surgery^b^(n = 69)	
	Baseline	Baseline	After 3 months
Dietitian consultation, No. (%)	36 (38%)	24 (35%)	40 (58%)
Missing	1 (1%)	1 (1%)	1 (1%)
Oral tube feeding, No. (%)	4 (4%)	4 (6%)	2 (3%)
Missing	1 (1%)	0 (0%)	2 (3%)
Oral nutritional supplements, No. (%)	28 (30%)	18 (26%)	26 (38%)
Reduced food intake, No. (%)	50 (53%)	35 (51%)	49 (71%)
Missing	1 (1%)	1 (1%)	0 (0%)
Self‐reported BMI, kg/m^2^, median (IQR)	25 (22, 27)	25 (21, 28)	23 (21, 27)
<18.5 (underweight)	6 (6%)	5 (7%)	8 (12%)
18.5–25 (normal weight)	45 (48%)	32 (46%)	35 (51%)
≥25 (overweight)	42 (45%)	31 (45%)	26 (38%)
Missing	1 (1%)	1 (1%)	0 (0.0%)
Weight loss at baseline^c^, No. (%)		NA	NA
<10%	66 (70%)		
≥10%	28 (30%)		
Cachexia, No. (%)	59 (63%)	41 (59%)	NA
Pancreatic enzyme supplementation, No. (%)	19 (20%)	13 (19%)	44 (64%)
Missing	2 (2%)	1 (1%)	5 (7%)
Protein score, No. (%)			
<10	62 (66%)	44 (64%)	47 (68%)
10	26 (28%)	20 (29%)	17 (25%)
Missing (did not complete DHD‐FFQ)	6 (6%)	5 (7%)	5 (7%)

Abbreviations: BMI, body mass index; NA, not applicable.

^a^ Baseline is before or within one week after start neoadjuvant chemotherapy.

^b^ After surgery is based on the 3 or 9 month questionnaire, depending on time of surgery.

^c^ Reported half year weight loss in baseline questionnaire.

Of all 70 patients who received palliative chemotherapy, 54 patients (77%) had cachexia at baseline (Table 3, Figure 2). Dietitian consultation was offered in 28 of these cachectic patients (52%). In patients with a BMI ≥25, both cachexia (33 patients (80%) vs. 21 patients (72%), respectively, *p* = 0.428) and weight loss ≥10% (21 patients (51%) vs. 10 patients (34%), respectively, *p* = 0.164) were not different compared to patients with a BMI <25. Of all 53 patients (76%) with an insufficient protein score at baseline, 17 (32%) had a dietetic consultation and 17 (32%) received oral nutritional supplements. Pancreatic enzymes were supplemented in 12 patients (23%) at baseline and this increased to 17 patients (32%) after 3 months.

**TABLE 3:** Nutritional and weight parameters in patients who received palliative chemotherapy

	Patients who completed the questionnaire at baseline^a^(n = 70)	Patients who completed the baseline and 3 month questionnaires (n = 53)	
	Baseline	Baseline	After 3 months
Dietitian consultation, No. (%)	29 (41%)	21 (40%)	21 (40%)
Missing	0 (0%)	0 (0%)	0 (0%)
Oral tube feeding, No. (%)	3 (4%)	2 (4%)	2 (4%)
Oral nutritional supplements, No. (%)	22 (31%)	15 (28%)	22 (42%)
Reduced food intake, No. (%)	55 (79%)	42 (79%)	33 (62%)
Self‐reported BMI, kg/m^2^, median (IQR)	25 (22, 26)	24 (22, 26)	23 (21, 25)
<18.5 (underweight)	1 (1%)	1 (2%)	3 (6%)
18.5–25 (normal weight)	40 (57%)	32 (60%)	36 (68%)
≥25 (overweight)	29 (41%)	20 (38%)	12 (23%)
Missing	0 (0%)	0 (0%)	2 (4%)
Weight loss at baseline^b^, No. (%)		N/A	N/A
<10%	39 (56%)		
≥10%	31 (44%)		
Cachexia, No. (%)	54 (77%)		N/A
Pancreatic enzyme supplementation, No. (%)	16 (23%)	12 (23%)	17 (32%)
Missing	4 (6%)	4 (8%)	5 (9%)
Protein score, No. (%)			
<10	53 (76%)	40 (75%)	37 (70%)
10	13 (19%)	10 (19%)	12 (23%)
Missing (did not complete DHD‐FFQ)	4 (6%)	3 (6%)	4 (8%)

Abbreviations: BMI, body mass index; N/A, not applicable.

^a^ Baseline is in patients who received palliative chemotherapy: before or within one week after start palliative chemotherapy.

^b^ Reported half year weight loss in baseline questionnaire.

Of all 38 patients with best supportive care 31 patients (82%) had cachexia of whom 22 had a dietetic consultation (71%) (Figure 2). Tube feeding and oral nutritional supplements were used in 5 patients (13%) and 16 patients (42%), respectively. None used parenteral nutrition.

### 3.2 Survival

**FIGURE 3:**
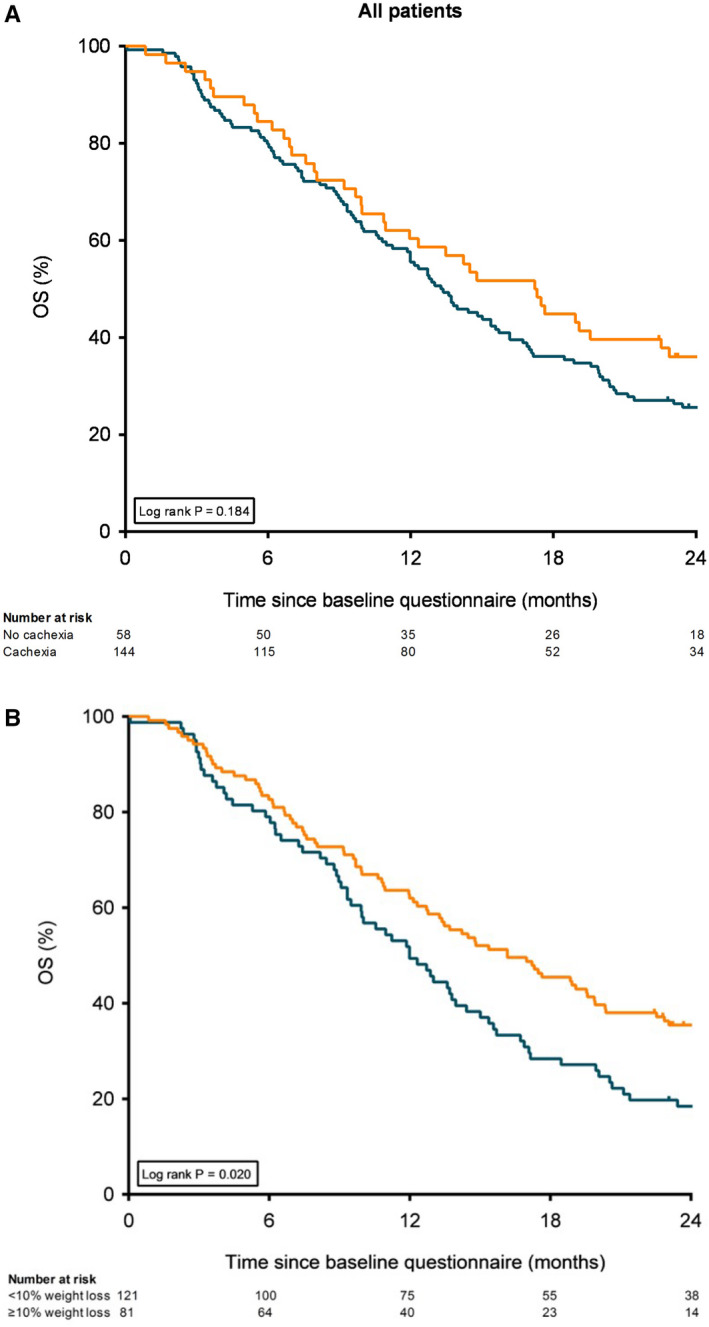
Kaplan–Meier curves showing overall survival for all patients. A, Kaplan–Meier curves stratified by cachexia at baseline. B, Kaplan–Meier curves stratified by weight loss (<10 or ≥10%) at baseline

Median overall survival in patients with cachexia was 13 months (IQR 11–16), which was not significantly different to patients without cachexia (17 months, IQR 11–23, *p* = 0.18, Figure 3A), although patients with cachexia tended to have a poorer survival. In patients with ≥10% weight loss median overall survival was significantly lower, as compared to those with <10% weight loss (12 months (IQR 7–20) vs. 16 months (IQR 8–31), *p* = 0.02, Figure 3B). Analyses in the subgroups based on treatment showed no differences in survival in patients with and without cachexia, and with <10% or ≥10% weight loss.

## 4 DISCUSSION

This prospective multicenter cohort study showed that 71% of patients with pancreatic cancer presented with cachexia at diagnosis of whom almost half had no dietetic consultation. Survival did not differ in patients with and without cachexia at diagnosis, but was shorter in patients with more severe weight loss (≥10%).

This is the first multicenter study to investigate the real‐life situation regarding cachexia and dietetic consultation in pancreatic cancer. In previous studies, cachexia was diagnosed in 31%–41% of patients with resectable pancreatic cancer, which is lower than in our study.^4^, ^10^This could be explained by different definitions. In our study, BMI was also taken into account, whereas in previous studies cachexia was defined as weight loss >10%.^4^, ^7^, ^10^We therefore also assessed weight loss ≥10%, which was 40% and thus, in line with previous studies. The high prevalence of cachexia underscores that physicians should be aware of cachexia in patients with pancreatic cancer.

In the three subgroups based on treatment for pancreatic cancer, patients undergoing best supportive care most often received dietitian support. In general, these patients were in their end‐of‐life, and therefore, the rate of dietetic consultation was higher than expected on forehand, while the low percentage of dietetic consultation in patients who underwent surgery is surprising. It could be speculated that this difference occurred because dietitian consultation is one of the few things that can be offered to patients with best supportive care. In addition, it should be noted that patients with best supportive care group are a selection of a relatively fit subgroup, since in general patients with a relatively good performance status are included in PACAP PROMs. It is possible that in these relatively fit patients, physicians may be more likely to start an intervention, including dietetic consultation.^25^However, in these patients in the end‐of‐life stage, dietitian support is not focusing on improving nutritional status anymore, but is tailored to the patients’ needs and wishes enhancing comfort and support quality of life.^26^In contrast, in patients with anticancer treatment, dietitian support may be considered less important by physicians compared to the impact of surgery or chemotherapy on survival. Another explanation could be under reportation by the patient, or nutritional support given by an experienced nurse practitioner. Dietitian counseling, however, could support the effects of anticancer treatments and might diminish complications by optimizing nutritional status.^14^, ^27^To optimize dietetic consultation, all patients with pancreatic cancer planned for life enhancing treatment should be screened for malnutrition.^28^Screening could be performed according to the GLIM criteria, which present a consensus scheme for diagnosing malnutrition in adults in clinical settings.^8^Based on our results, it could be advised to include the screening for malnutrition in standard operating procedures, because the referral rate could be greatly improved.

It was expected that cachexia would negatively affect overall survival, but patients with and without cachexia at baseline did not show differences in overall survival. Nevertheless, patients with more severe weight loss (≥10%) had a decreased survival compared to those with <10% weight loss. This is also shown in previous studies.^29^, ^30^A higher percentage of weight loss, especially in combination with a lower BMI, independently decreases survival.^30^This could partially explain the difference in results between cachexia and severe weight loss. Moreover, the study might be underpowered for demonstrating a significant effect in patients with cachexia, because both in patients with cachexia as well as in those with severe weight loss, survival is decreased by 4 months. It could be suggested that an early start of nutritional interventions in patients with cachexia could prevent the development of severe weight loss and consequently improve survival. It should be mentioned that survival is also affected by other factors, such as tumor location, tumor stage, postoperative complications, use of (adjuvant) chemotherapy, and disease recurrence.^31^, ^32^Prevention of weight loss only will probably not result in a clinically relevant improvement of survival.

In more than half of patients who underwent surgery and who received palliative chemotherapy, the protein score was <10. This already indicates that protein intake should be improved because the score for protein intake has been based on cut‐offs for the general healthy population. Since this study is focusing on pancreatic cancer patients, in whom it is even recommended to use a high protein diet, all patients with a score <10 should have a dietetic consultation.^28^In our cohort, 39% of the patients with a protein score <10 had a dietetic consultation and around one‐third used oral nutritional supplements. This demonstrates that there is room for improvement. After referral, a dietitian will devise a patient tailored nutritional support plan based on the patient's nutritional intake to improve nutritional status in patients undergoing surgery or palliative chemotherapy. In patients receiving best supportive care, dietary support should be focused at comfort and increasing quality of life and should not be based on improving protein intake.

Another important aspect of malnutrition is exocrine pancreatic insufficiency.^33^, ^34^, ^35^, ^36^In patients with pancreatic cancer, exocrine insufficiency is highly prevalent, but currently underdiagnosed and undertreated.^33^, ^34^, ^37^Enzyme supplementation was not reported in more than a quarter of the patients that received palliative chemotherapy, which is low compared to the prevalence of exocrine insufficiency at diagnosis (66%).^34^Nearly two‐thirds of patients who underwent surgery, however, received enzyme supplementation after surgery, probably because it was included in hospitals’ postoperative protocols.^11^This could also be increased as the incidence of exocrine insufficiency in these patients is 74% at 6 months postoperatively.^33^Enzyme supplementation requires more attention and also support from a dietitian or nurse practitioner to provide patient education on proper use of enzymes.^37^, ^38^

The strength of this prospective multicenter study is the real‐life overview of the proportion of patients with cachexia and the current situation regarding dietetic consultation. The study also had some limitations. First, selection bias may be present toward a relatively high proportion of patients who underwent resection and/or received adjuvant or palliative chemotherapy.^31^Possibly, fitter patients were more keen on being included, which might have led to an underestimation of malnutrition in our cohort. This emphasizes the need for proper identification and treatment of malnutrition even more. Second, nutritional support based on dietetic consultation could be underestimated, because of an under reportation of patients or support by a nurse practitioner. In large and specialized pancreatic surgery centers, an important role is often reserved for nurse practitioner or physician assistants experienced within pancreatic cancer to consult patients on their nutritional status and pancreatic enzyme supplementation. Third, the DHD‐FFQ was included in the PROMs to assess the diet quality according (and derived protein score) to the national dietary guideline, but was only a qualitative measure and not quantitative. Fourth, patients, especially those who underwent surgery, frequently did not complete the baseline questionnaire before start of treatment which unfortunately resulted in exclusion of these patients and might be prevented by improved logistics within PACAP. Fifth, the criterion for cachexia that included the presence of sarcopenia was not used, because these data were not available in the NCR or PROMs.^7^The loss of skeletal muscle mass depletion is the main aspect of malnutrition that predicts the risk of physical impairment, complications and mortality, and probably led to an underestimation of patients with cachexia.^28^, ^39^, ^40^However, some advocate to focus on cachexia rather than sarcopenia in pancreatic cancer, because cachexia is the main problem.^41^

In conclusion, this study showed that over two‐thirds of patients with pancreatic cancer present with cachexia of which nearly half had no dietetic consultation. Only 39% of patients with a protein score <10 had a dietetic consultation and pancreatic enzyme supplementation could be increased. Survival was comparable in patients with and without cachexia, but significantly decreased in patients with severe weight loss (≥10%) suggesting the importance of preventing further weight loss. Increased awareness of cachexia and severe weight loss, screening on (the risk) of malnutrition based on the GLIM criteria, and dietetic consultation to improve protein intake could be helpful improving treatment outcomes.

## CONFLICT OF INTEREST

AL has received research funding from Mylan and Allergan. MvO has received unrestricted research grants from BMS, Merck Serono, Nordic, Roche, and Servier. JdV has received non‐financial support and institutional research funding from Servier, outside the submitted work. HvL has served as a consultant for BMS, Celgene, Lilly, Nordic, and Servier, and has received unrestricted research funding from Bayer, BMS, Celgene, Lilly, Merck Serono, MSD, Nordic, Philips, Roche, and Servier. IdH has received an unrestricted research fund paid to the institute from QPS/RanD, ROCHE, and KWF for unrelated research. The other authors have nothing to disclose.

## AUTHORS’ CONTRIBUTION

Study design: All co‐authors. Data acquisition: AL, WD, TM. Data analysis and interpretation: AL, WD, TM, SB, MdvdS, MB, HvL. Manuscript writing: AL, WD, TM, SB, MdvdS, MB, HvL. Manuscript review: all co‐authors. Final approval: all co‐authors.

## ETHICAL STATEMENT

The Dutch Pancreatic Cancer Project (PACAP) was judged by the Medical Ethics Review Committee of the AMC and did not require an official approval by this committee as the Medical Research Involving Human Subject Act did not apply for this study. All patients provided written informed consent for participation in the PROMs and linkage of data from the PROMs and NCR.

## Data Availability

The data that support the findings of this study are available from the NCR and PACAP and might be available on request from the corresponding author. The data are not publicly available due to privacy or ethical restrictions. Restrictions apply to the availability of these data, which were used under license for this study, and requests will be discussed by the principal investigators.
